# Extracellular histones aggravate autoimmune arthritis by lytic cell death

**DOI:** 10.3389/fimmu.2022.961197

**Published:** 2022-08-11

**Authors:** Jaeyong Jung, Lucy Eunju Lee, Hanna Kim, Ji Eun Kim, Sung Hoon Jang, Jong Seong Roh, Beomgu Lee, William H. Robinson, Dong Hyun Sohn, Jae-Chul Pyun, Jason Jungsik Song

**Affiliations:** ^1^ Department of Materials Science and Engineering, Yonsei University, Seoul, South Korea; ^2^ Division of Rheumatology, Department of Internal Medicine, Dongguk University Ilsan Hospital, Goyang, South Korea; ^3^ Division of Rheumatology, Department of Internal Medicine, Yonsei University College of Medicine, Seoul, South Korea; ^4^ Department of Herbal Prescription, College of Korean Medicine, Daegu Haany University, Gyeongsan, South Korea; ^5^ Department of Microbiology and Immunology, Pusan National University School of Medicine, Yangsan, South Korea; ^6^ VA Palo Alto Health Care System, Palo Alto, CA, United States; ^7^ Division of Immunology and Rheumatology, Stanford University, Stanford, CA, United States; ^8^ Institute for Immunology and Immunological Diseases, Yonsei University College of Medicine, Seoul, South Korea

**Keywords:** histone, cytotoxicity, DAMP, chemokine, synoviocyte, macrophage, inflammation, arthritis

## Abstract

Although recent studies have demonstrated a proinflammatory effect of extracellular histones in sepsis *via* endothelial cytotoxicity, little is known about their contribution to autoimmune arthritis. Therefore, we investigated the role of extracellular histones in autoimmune arthritis and their cytotoxic effect on synoviocytes and macrophages. We measured histones in the synovial fluid of patients with rheumatoid arthritis (RA) and evaluated arthritis severity in a serum-transfer arthritis (STA) mouse model with intraperitoneal histone injection. Histone-induced cytotoxicity was measured using SYTOX green staining in the synoviocyte cell line MH7A and macrophages differentiated from the monocytic cell line THP-1, and the production of damage-associated molecular patterns (DAMPs) was measured by HMGB1 and ATP. Furthermore, we performed RNA-seq analysis of THP-1 cells stimulated with H2B-α1 peptide or with its citrullinated form. The levels of histones were elevated in RA synovial fluid, and histones aggravated arthritis in the STA model. Histones induced cytotoxicity and DAMP production in synoviocytes and macrophages. Chondroitin sulfate reduced histone-induced cytotoxicity, while lipopolysaccharides aggravated cytotoxicity. Moreover, the cytotoxicity decreased when the arginines in H2B-α1 were replaced with citrullines, which demonstrated its electrostatic nature. In transcriptome analysis, H2B-α1 changed the gene expression pattern of THP-1 cells involving chemokines, interleukin-1, -4, -10, -13, and toll-like receptor (TLR) signaling pathways. Extracellular histones were increased in RA synovial fluid and aggravated synovitis in STA. They induced lytic cell death through electrostatic interaction with synoviocytes and macrophages, leading to the secretion of DAMPs. These findings suggest that histones play a central role in autoimmune arthritis.

## Introduction

Histones are DNA-binding proteins that constitute nucleosomes, the basic subunits of chromatin. The four classes of core histones (H2A, H2B, H3, and H4) have similar structures, with a preserved histone fold domain and an unstructured amino acid tail (1). Positively charged histones and negatively charged phosphate groups of DNA combine to form a stable structure (2). In addition to their function in the nucleus, studies have highlighted that DNA–histone complexes are released into the extracellular space during cell death processes like necrosis and NETosis (3, 4).

NETosis is one of the lytic forms of cell death that releases neutrophil extracellular traps (NETs) that can capture and kill pathogens (5). The immune response against DNA, histones, and other proteins associated with NETs can be induced to form autoantibodies (6–8), and these intracellular components can induce inflammatory responses (9, 10). Histones, which comprise 70% of the protein components of NETs (9), cause cell injury and death in endothelial cells (11) and organ failure when released into the extracellular space (12). Proinflammatory properties of extracellular histone have been demonstrated in sepsis (4), chronic obstructive pulmonary disease (COPD) (13), systemic lupus erythematosus (14), disseminated intravascular coagulation (DIC) (15), and malignancies (16, 17), but the molecular mechanisms have not been well understood.

Rheumatoid arthritis (RA) is characterized by chronic synovial inflammation with the detection of autoantibodies that recognize citrullinated proteins (18). Recent studies have shown the role of citrullinated histones as autoantigens in RA (19). Activation of PAD4 gives rise to citrullinated histones, making NETosis a key driver of autoantigen production in RA (20, 21). However, the role of extracellular histones in RA remains largely unexplored. In this study, we investigated the influence of histones released from synovial cells during cell death processes on synovial inflammation.

## Materials and methods

### Determination of extracellular histones in synovial fluid samples

Human synovial fluid samples were obtained under a protocol for discarded specimens approved by the Stanford University School of Medicine Institutional Review Boards that does not require consent. The samples were stored frozen at -80 °C until the time of analysis. Patients with RA that met the American College of Rheumatology criteria for the disease were included in the study (22). The sample donors were patients with knee osteoarthritis (OA) and over 45 years of age with radiographic Kellgren/Lawrence grade ≥ 3 changes (23). The levels of H2A, H2B, H3, and H4 were determined using the enzyme-linked immunosorbent assay (ELISA) kit for each histone (antibodies-online, Aachen, Germany) according to the manufacturer’s protocol.

### K/BxN serum-transfer arthritis (STA) mouse model

Eight-week-old male C57BL/6 mice (Koatech, Pyeongtaek, Korea) were housed under controlled conditions at 23 ± 2°C and 50 ± 10% humidity on a 12 h light/dark cycle and had free access to a standard chow diet and water. STA was induced *via* intraperitoneal injection of 150 μL of K/BxN serum into C57BL/6 mice on day 0 and day 2. The mice were intraperitoneally injected with 200 μg of calf histone (Roche, Basel, Switzerland) or phosphate-buffered saline on day 1 and day 3. We used native histones purified by calf thymus. It is a mixture of histones H1, H2A, H2B, H3, and H4, and we used it as it is without any additional procedures. The histone treatment dose for intraperitoneal injection was determined based on the previously published method in a sepsis model, which demonstrated that the intravenously injected calf thymus histones were lethal at a concentration of 75-100 mg/kg and sublethal at 50 mg/kg in C57BL/6 mice (12). To investigate the effect of histones in arthritis, we injected 200 μg of calf thymus histones (equivalent to 10 mg/kg) intraperitoneally into each mouse based on our preliminary dose titration study (data not shown). The clinical arthritis score was determined as previously described (19): 0, no swelling or erythema; 1, mild swelling and erythema or digit inflammation; 2, moderate swelling and erythema confined to the region distal to the mid-paw; 3, pronounced swelling and erythema with extension to the ankle; and 4, severe swelling, erythema, and joint rigidity of the ankle, foot, and digits. The severity of arthritis in each limb was graded on a scale of 0–4 (a maximum possible score of 16 for each mouse) by a nonblinded examiner. Hind paw thickness was measured with a dial thickness gauge. The mouse joint tissues were fixed with 10% formalin, decalcified in 5% formic acid, and embedded in paraffin. Sections of the paraffin-embedded tissue were stained with hematoxylin and eosin (H&E), and scored by a blinded examiner for synovitis, pannus formation, and erosion of bone and/or cartilage based on a previously described scoring system (24). For each experimental condition, the joints of ten mice were evaluated and scored. The sample size was determined based on our pilot experiments. Mice were assigned to each group at random, and the order of treatments was randomized. All mice were included in the analysis. All animal experiments and protocols were approved by the Institutional Animal Care and Use Committee at Pusan National University (Miryang, Korea) and performed in accordance with the institutional and national guidelines for the care and use of laboratory animals.

### Cell culture and reagents

Human synovial fibroblast cell line MH7A was cultured in RPMI-1640 (Hyclone, Logan, UT, USA) containing 25 mM HEPES and L-glutamine (Hyclone), supplemented with heat-inactivated 10% fetal bovine serum (FBS; Hyclone), 100 units/mL penicillin (Hyclone), and 100 μg/mL streptomycin (Hyclone). THP1-HMGB1-Lucia cells (THP-1 cells, InvivoGen, San Diego, CA, USA) were cultured in RPMI-1640, supplemented with 25 mM HEPES, L-glutamate, heat-activated 10% FBS, 100 units/mL penicillin, 100 μg/mL streptomycin, and 100 μg/mL Normocin (InvivoGen). For all assays, THP-1 cells were seeded at a density of 1 × 10^5^ cells/well in 96-well plates and differentiated into adherent macrophages with 25 ng/mL phorbol 12-myristate 13-acetate (PMA, Sigma-Aldrich, MO, USA) overnight. Cells were treated with calf thymus histones (Abnova, Taipei, Taiwan) or recombinant histones, H2A, H2B, H3, and H4 (New England BioLabs, Cambridge, MA, USA) or histone peptides (Peptron, Daejeon, Korea, **Table 1**).

**Table 1:** Sequences of histone peptides.

Histone peptides	Sequence
H2A-α1	KTRSSRAGLQFPVGRVHRLLRKGNYSERVGAGAP
H2B-α1	QKKDGKKRKRSRKESYSIYVYKVLKQVHPDTGIS
cit-H2B-α1	QKKDGKK-Cit-K-Cit-S-Cit-KESYSIYVYKVLKQVHPDTGIS
H3-α1	LIRKLPFQRLVREIAQDFKTDLRFQ
H4-α1	RHRKVLRDNIQGITKPAIRRLARRGGVKRISGLI

### Lactate dehydrogenase (LDH) release assay

The level of LDH released into the medium from cells was measured using an LDH assay kit (DoGenBio, Seoul, Korea). This kit measures the amount of a water-soluble tetrazolium salt (WST) to determine the cytotoxicity of cells. Cells were seeded onto 96-well plates at a density of 1 × 10^5^ cells/well in serum-free media. After incubation with different concentrations of histones, 10 μL supernatant of each sample and 100 μL LDH Reaction Mix (WST substrate mix, LDH assay buffer) were incubated at 25°C in the dark for 30 min, and the optical density of soluble WST was then measured at 450 nm using FlexStation3 microplate reader (Molecular Devices, San Jose, CA, USA).

### Fluorescence imaging using SYTOX Green dead cell stain

MH7A cells and THP-1 cells were placed at a density of 1 × 10^5^ cells/well and 5 × 10^5^ cells/well, respectively. The cells were treated with 5 μM Hoechst 33342 stain (ThermoFisher Scientific, Waltham, MA, USA) for 1 h, then treated with various concentrations of histones and/or lipopolysaccharide (LPS, Sigma-Aldrich) and stained with a plasma membrane-impermeable DNA-binding dye, SYTOX Green (Invitrogen), according to the manufacturer’s protocol, in a serum-free condition. Before histone addition, the cells were treated with or without 10 mM sodium perchlorate (Sigma-Aldrich) for 24 h. The live images of the cells were obtained under a fluorescence microscope (Eclipse Ti2, Nikon Instruments, Tokyo, Japan) or by an automatic cell imaging system (Operetta CLS™, PerkinElmer, Waltham, MA, USA). For images obtained from the Operetta cell imaging system, image analysis and cell counting were performed according to the manufacturer’s assay protocol using Harmony^®^ software (Version 4.9, PerkinElmer).

### Quantification of HMGB1 *via* luciferase assay

The level of HMGB1::Lucia luciferase released from THP-1 cells was measured with a luminometer (FlexStation3) using luciferin. A sample from each well was mixed with QUANTI-Luc (InvivoGen) according to the manufacturer’s instructions, and the luciferase reading was measured.

### Adenosine triphosphate (ATP)-monitoring luminescence assay

ATPlite (PerkinElmer) is a luciferase-based assay used for the quantitative evaluation of cell proliferation and cytotoxicity. For this assay, cells were seeded onto a 96-well plate at a density of 5 × 10^5^ cells/well. The cells were treated with different concentrations of histones for 1 h. Then, the ATPlite buffer and the lyophilized substrate solution were mixed. Finally, cells in each well were incubated with 50 μL of this mixture for 10 min, followed by a luminescence measurement.

### Fluorescence imaging to measure poly-L-lysine

?A3B2 tlsb .2pt?>To measure the cellular binding of FITC labeled poly-L-lysine (Sigma-Aldrich), THP-1 cells were seeded at a density of 2 × 10^5^ cells/well and treated with 100 ng/mL LPS for 24 h. Then, 10 μg/mL poly-L-lysine-FITC was added to the cells and incubated for 30 min at 4°C. Cells were observed under a fluorescence microscope (Eclipse Ti2) to obtain fluorescence images. Quantification of fluorescence intensity of images was performed using ImageJ (https://imagej.nih.gov).

### Flow cytometry to measure cell viability using 7-aminoactinomycin D (7-AAD)

To detect cell membrane damage in flow cytometry, a DNA-staining fluorescent marker 7-AAD (Invitrogen) was used. MH7A and THP-1 cells were seeded at a density of 5 × 10^5^ cells/well and treated with various concentrations of histones for 1 h. Subsequently, 1 μg/mL 7-AAD was added to the cells and incubated for 30 min. Cells were analyzed on BD LSRII SORP (BD Biosciences, San Jose, CA, USA) to detect 7-AAD.

### ELISA for Interleukin-1β (IL-1β), tumor necrosis factor-alpha (TNF-α), and C-C motif chemokine ligand 4 (CCL4)

IL-1β (eBioscience, San Diego, CA, USA), TNF-α (Invitrogen, San Diego, CA, USA), and CCL4 (R&D Systems, Minneapolis, MN, USA) levels were measured using ELISA kits as per the manufacturer’s instructions.

### RNA-seq and analysis of differentially expressed genes (DEGs)

Total RNA was isolated from THP-1 cells treated with H2B-α1 peptide or cit-H2B-α1 peptide, using TRIzol reagent (Invitrogen). Samples of 3 μg of total RNA were prepared from each condition with three experimental replicates, and gene expression profiling was performed using the QuantSeq 3′ mRNA-Seq service (Ebiogen, Seoul, Korea). After quality checks using Bioanalyzer 2100 system (Agilent, Santa Clara, CA, USA), an oligo-dT primer set containing Illumina-compatible sequences at its 5′ end was hybridized to the prepared RNA, and reverse transcription was performed. High-throughput sequencing was performed as single-end sequencing using Illumina NextSeq 500 (San Diego, CA, USA). The resulting reads were aligned using Bowtie2 (http://bowtie-bio.sourceforge.net/bowtie2) with the human reference genome hg19 (25). The DEGs were determined based on the counts from alignments using coverage in Bedtools (https://bedtools.readthedocs.io). The read count data were processed based on the quantile normalization method using EdgeR (https://bioconductor.org). Gene enrichment analysis was performed by MATLAB (MathWorks, Natick, MA, USA) using the Reactome pathway database (https://reactome.org) and Gene Ontology Consortium database (http://geneontology.org).

### Statistical analysis

Statistical analysis was performed using Prism 9 software (GraphPad, San Diego, CA, USA). Data are presented as mean ± standard error of the mean or median ± interquartile range. In addition, we performed an unpaired t-test or Mann–Whitney test to analyze the statistical differences between the two groups. One-way or two-way ANOVA with Tukey–Kramer multiple comparison tests were used for comparisons between multiple groups. Statistical significance level was set at *P* < 0.05.

## Results

### Histones are increased in RA synovial fluid and aggravate arthritis in STA mice

**Figure 1:**
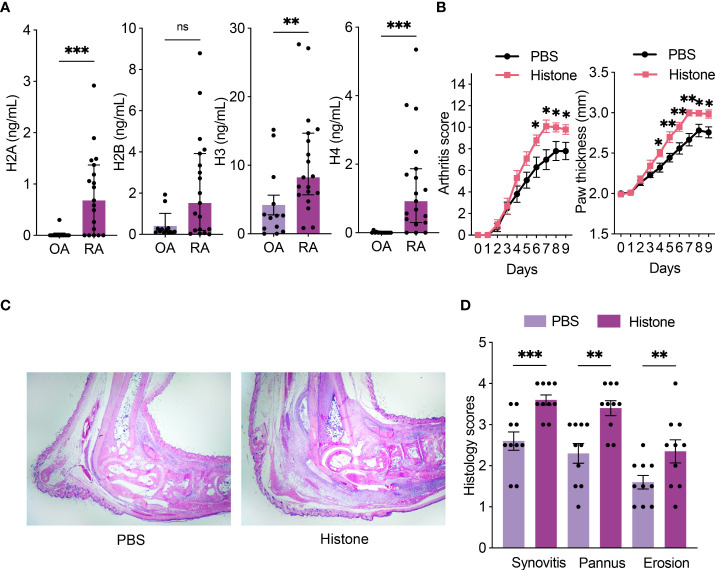
Histones are increased in RA synovial fluid and aggravate arthritis in STA mice. **(A)** Levels of histones (H2A, H2B, H3, and H4) in synovial fluid from osteoarthritis (OA; n = 13) and rheumatoid arthritis (RA; n = 19) patients. **(B)** Serum-transfer arthritis (STA) severity and paw thickness in mice (n = 10 per group) with intraperitoneal histone or phosphate-buffered saline (PBS) injection. K/BxN serum was given on days 0 and 2, and 200 μg of histone or PBS was given on days 1 and 3. **(C)** Representative images of hematoxylin and eosin (H&E) stained sections of ankle joints (×20 magnification). **(D)** Histologic scores of synovitis, pannus formation, and erosions of ankle joints. Data are shown as the median ± interquartile range **(A)** or the mean ± standard error of the mean (B and D). The *P*-values were calculated by Mann–Whitney tests **(A)** or unpaired t-tests **(B, D)**. *P < 0.05, **P < 0.01, and ***P < 0.001. ns, not significant..

The levels of histones H2A, H2B, H3, and H4 were higher in RA compared to those in OA synovial fluid samples, though the difference in H2B was not statistically significant **(** **Figure 1A** **)**. We tested whether extracellular histones could aggravate inflammatory arthritis. When mice were induced for STA, co-injection of histone aggravated arthritis **(** **Figure 1B** **)**. Histopathological analyses also showed that histones increased synovitis, pannus formation, and erosion of bone and cartilage **(** **Figures 1C, D** **)**. These results showed that histones have a deleterious effect on arthritis.

### Histones induce cytotoxicity in synoviocytes and macrophages

**Figure 2:**
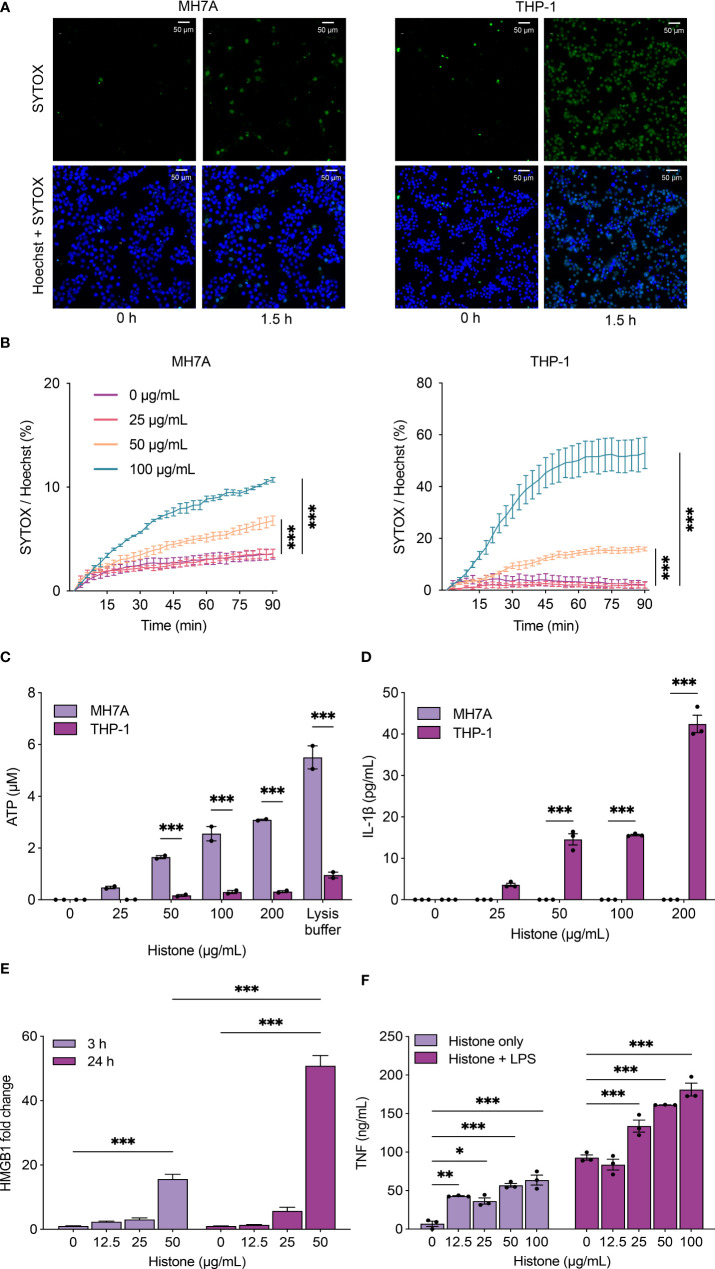
Histones induce cytotoxicity and the release of damage-associated molecular patterns (DAMPs). **(A)** Histone-induced cytotoxicity in MH7A and THP-1 cells detected by SYTOX Green. Representative fluorescence microscopy images of MH7A (left) and THP-1 cells (right) with 100 μg/mL of histones. See also **Supplement Movies 1A**, **B** **(B)** Time-course plot of dead cells detected by SYTOX green with the Operetta cell imaging system, MH7A (left) and THP-1 cells (right). **(C)** Levels of extracellular ATP from MH7A and THP-1 cells treated with histones for 1 h. **(D)** Levels of extracellular IL-1β from MH7A and THP-1 cells treated with histones for 1 h. **(E)** Levels of HMGB1 from THP-1 cells treated with histones for 3 h or 24 h. **(F)** Levels of TNF-α from THP-1 cells treated with histones and/or lipopolysaccharides (LPS; 10 ng/mL) for 24 h. Data are expressed as the mean ± standard error of the mean. ANOVA with Tukey–Kramer multiple comparison tests were conducted. *P < 0.05, **P < 0.01, and ***P < 0.001. The experiments were performed with technical replicates of three wells per condition.

We investigated if the extracellular histones could induce cell injury and death in synoviocytes and macrophages. We observed that histones increased cytotoxicity in MH7A (synoviocytes) and PMA-differentiated THP-1 (macrophages) cells, as visualized by SYTOX Green dead cell staining (**Figures 2A, B** **;** **Supplementary Movies 1A** **and** **B**). Notably, histone-induced lytic cell death was more rapid and prominent in THP-1 cells than in MH7A cells **(** **Supplementary Figures S1, S2** **)**.

### Histones induce the release of DAMPs and cytokines

Synovial macrophages are one of the most abundant inflammatory cell types in the synovium of RA patients, and activated macrophages produce various cytokines and chemokines to recruit immune cells (26, 27). To investigate the mechanism of histone-induced cytotoxicity and its consequence in inflamed joints, we determined the level of ATP, one of the molecules released in cell damage. Extracellular ATP, which can act as a DAMP, was increased by histones in both MH7A cells and THP-1 cells (**Figure 2C**). In contrast, IL-1β was induced by histones only in THP-1 cells, not in MH7A cells (**Figure 2D**). The level of HMGB1, a well-studied DAMP, was also increased by histones in THP-1 cells (**Figure 2E**). We also measured the level of TNF-α when THP-1 cells were treated by histones with or without LPS. The increased level of TNF-α under histone-treatment demonstrated that histones cause a pro-inflammatory effect that is additive with LPS (**Figure 2F**).

### Chondroitin sulfate inhibits histone-induced cytotoxicity

**Figure 3:**
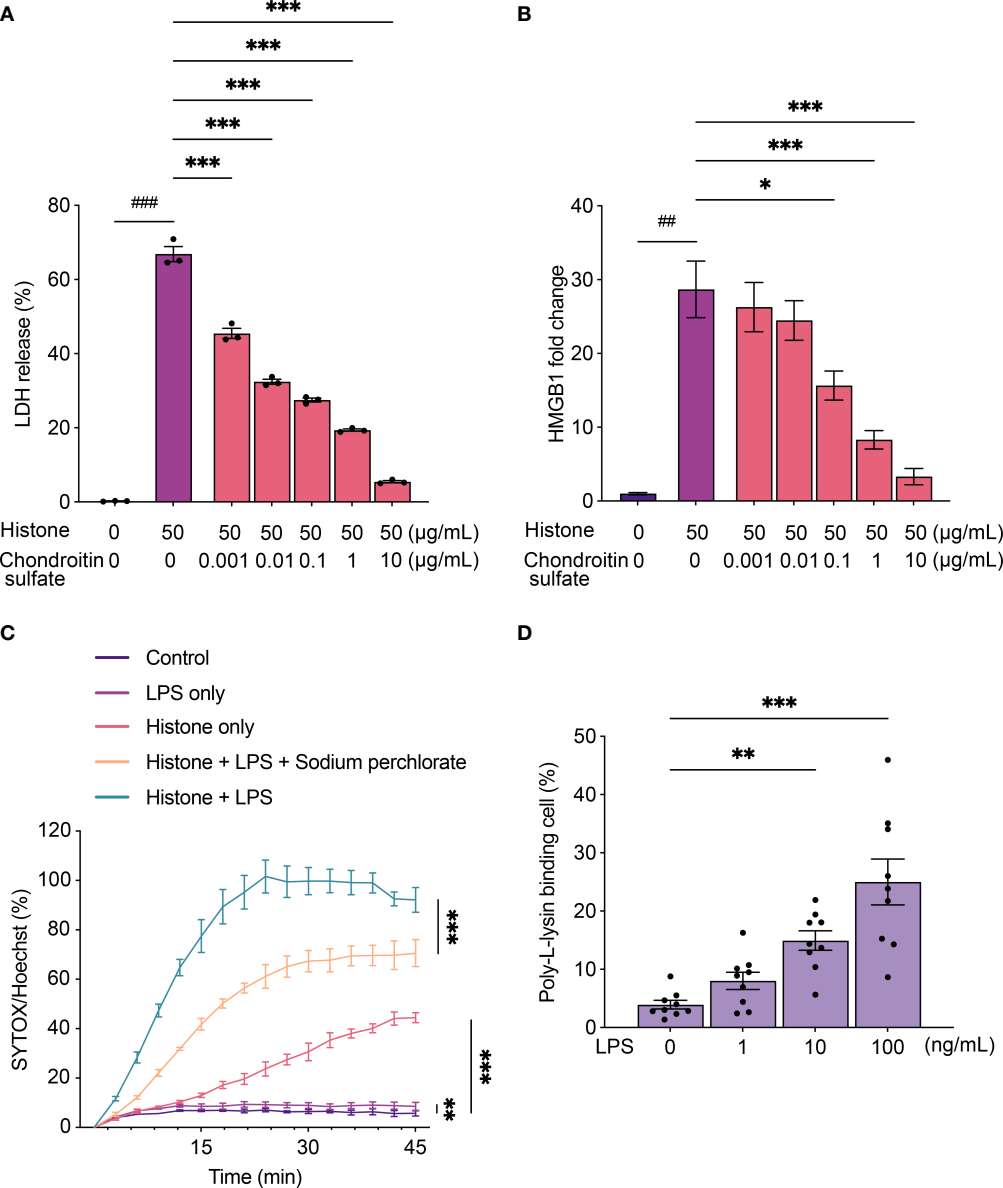
Chondroitin sulfate and lipopolysaccharides (LPS) regulate histone-induced cytotoxicity. **(A)** Lactate dehydrogenase (LDH) release assay using THP-1 cells to monitor histone-induced cytotoxicity for 24 h with chondroitin sulfate. **(B)** Levels of HMGB1 from THP-1 cells to monitor histone-induced cytotoxicity for 24 h with chondroitin sulfate. **(C)** Time-course plot of dead cells stained with SYTOX green evaluated by the Operetta cell imaging system after treatment with histone (100 μg/mL) and LPS (200 ng/mL) with sodium perchlorate (10 mM). **(D)** Poly-L-lysine binding to LPS-stimulated THP-1 cells. Data are expressed as the mean ± standard error of the mean. An unpaired t-test or a one-way ANOVA **(A, B)** or a two-way ANOVA **(C, D)** with Tukey–Kramer multiple comparison tests were conducted. *P < 0.05, **P < 0.01, and ***P < 0.001. ^##^ *P* < 0.01, and ^###^ *P* < 0.001. Different statistical tests were presented with different symbols.. The experiments were performed with technical replicates of three wells per condition.

Histones exhibit cytotoxicity by binding to cells through their strong positive charge (28). Since anionic polysaccharides and peptides neutralize the cytotoxicity of histones (29), we tested if chondroitin sulfate, one of the charged molecules in synovial fluid, could attenuate histone-induced cytotoxicity. Histone-induced cytotoxicity and HMGB1 production were strongly inhibited by chondroitin sulfate (**Figures 3A, B**).

### LPS increases the ability of histones to induce cytotoxicity

We also analyzed the effect of histones on cells treated with LPS. SYTOX Green staining of THP-1 cells revealed that histone-induced cytotoxicity increased when cells were treated with LPS and histones than with histones alone (**Figure 3C**). To verify the electrostatic interaction during the inflammatory process of THP-1 cells, we evaluated the binding of positively charged poly-L-lysine to cells when they were stimulated by LPS. When treated with LPS, increased poly-L-lysine binding was observed (**Figure 3D**), and treatment with sodium perchlorate, a sulfation inhibitor, reduced the cytotoxicity (**Figure 3C**).

### H2B-α1 peptide induces cytotoxicity

**Figure 4:**
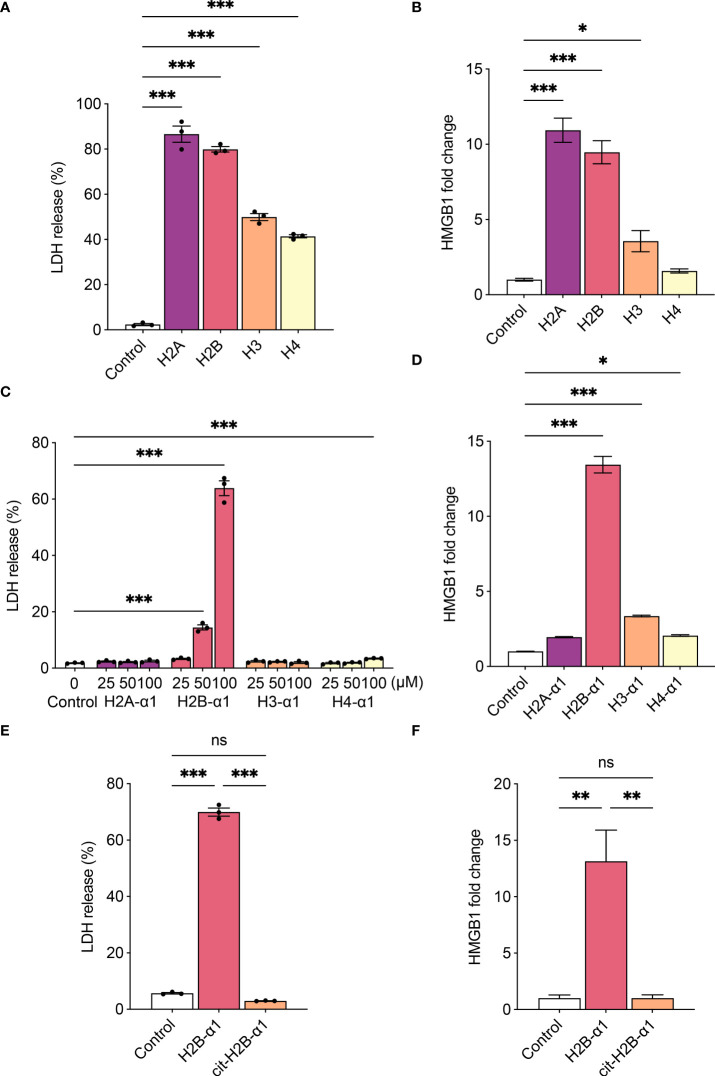
H2B-α1 peptide induces cytotoxicity in macrophage. **(A)**, **(B)** Histone-induced cytotoxicity (3 h) with each class of recombinant histone proteins (4 μM). **(C)**, **(D)** Histone-induced cytotoxicity (24 h) with each class of histone α1 peptides. **(E)**, **(F)** Histone-induced cytotoxicity (24 h) with native or citrullinated (cit) H2B-α1 peptide (50 μM). Cytotoxicity levels were measured by lactate dehydrogenase (LDH) release assay **(A, C, E)**, and HMGB1 release was measured by luciferase assay **(B, D, F)**. Data are expressed as the mean ± standard error of the mean. ANOVA with Tukey–Kramer multiple comparison tests were conducted. *P < 0.05, **P < 0.01, and ***P < 0.001. ns, not significant.. The experiments were performed with technical replicates of three wells per condition. See table 1 for sequences of peptides.

We investigated the classes of histones to reveal those that induce cytotoxicity. When THP-1 cells were treated with each histone, all histones induced cytotoxicity (**Figure 4A**), and all significantly increased HMGB1 production except for H4 (**Figure 4B**). As the recombinant histones were purified from *E. coli*, the levels of LPS in histones were measured and a significant amount of LPS was detected in H2B and H3 (**Supplementary Figure S3**). Since the LPS content of each histone was not proportional to the levels of LDH or HMGB1, LPS is not the major source of the cytotoxicity of the histones. However, the additive effect of the contaminated LPS in varying amounts should be considered. Referring to the previously studied histone structure (30, 31), we tested the cytotoxicity of α1 domain of each protein, H2A, H2B, H3, and H4 (**Table 1**). It has been suggested that histones bind to phospholipid-phosphodiester bonds using their DNA-binding α1 helices (32). When cells were treated with the α1 domain peptide of each histone, H2B-α1 peptide strongly induced cytotoxicity and HMGB1 production in THP-1 cells (**Figures 4C, D**). Since the binding of histones can be altered by modification of their positive charge, we replaced the positively charged arginines in H2B-α1 with citrullines (**Table 1**). Citrullination of H2B-α1 inhibited H2B-α1-induced cytotoxicity and HMGB1 production (**Figures 4E, F**).

### H2B-α1 peptide induces chemokine activation and citrullination alleviates the process

**Figure 5:**
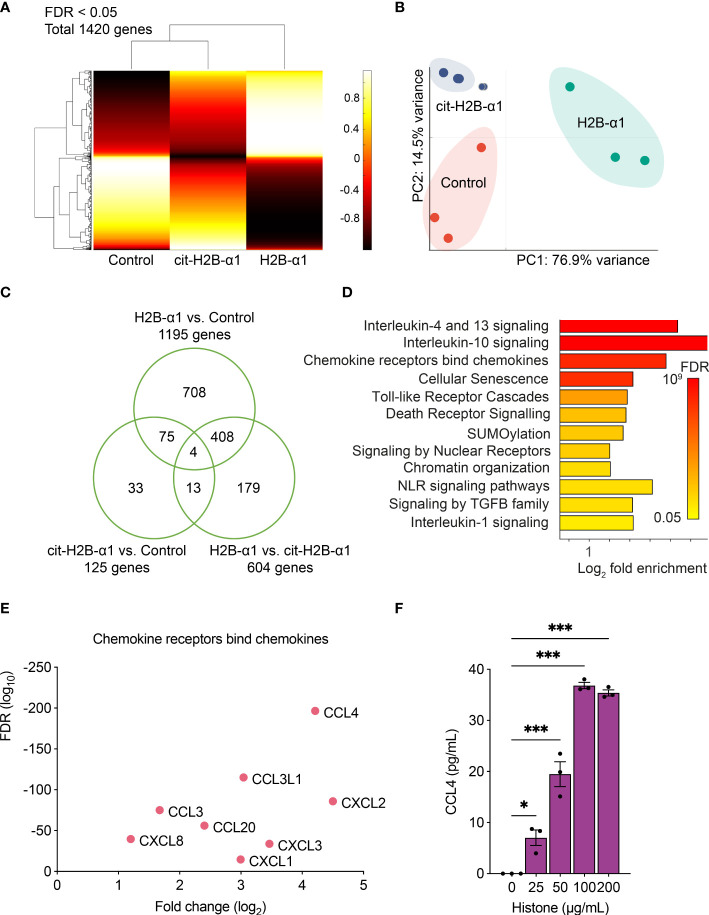
Transcriptome analysis of THP-1 cells with H2B-α1 peptide. **(A)** Expression clustering of RNA-seq data of THP-1 cells treated with H2B-α1 peptide or cit-H2B-α1 peptide for 3 h. **(B)** Principal component analysis of differentially expressed genes (DEGs). **(C)** Venn diagram of DEGs. **(D)** Significant gene ontology pathways from DEGs. **(E)** Top DEGs in chemokine pathway. **(F)** Levels of CCL4 with histone stimulation on THP-1 cells. Data in **(F)** are expressed as the mean ± standard error of the mean. ANOVA with Tukey–Kramer multiple comparison tests were conducted. *P < 0.05 and ***P < 0.001. The experiments were performed with technical replicates of three wells per condition.

To examine the cellular effects of histones, we analyzed the RNA-seq data of THP-1 cells treated with native or citrullinated H2B-α1 peptide. The clustergram representing the normalized expression of DEGs is shown (**Figure 5A**). Compared to THP-1 cells treated with H2B-α1 peptide, the transcriptome of the cells treated with citrullinated H2B-α1 peptide did not shift considerably from the expression pattern of the non-treated control cells. Applying principal component analysis (PCA) on 1420 DEGs, we verified two principal components, PC1 and PC2, explaining 76.9% and 14.5% of the variances, respectively (**Figure 5B**). In the two-dimensional PC space, representations of three replicates of each treatment group were exclusively distributed and completely separated. Transcriptome data was summarized as a Venn diagram (**Figure 5C**).

Gene ontology and pathway analysis were performed to examine the molecular pathways related to the effect of native histones. The most enriched Reactome pathways are listed in **Figure 5D**, and for each pathway, the top DEGs are shown based on the false discovery rate (FDR) and expression fold change (**Figure 5E** and **Supplementary Figures S4A–K**) (33). H2B-α1 peptide stimulation changed the gene expression pattern of THP-1 cells involving chemokines, IL-1, -4, -10, -13 signaling, and TLR signaling pathways. Citrullination of positively charged arginines of cytotoxic H2B-α1 peptide decreased histone-induced proinflammatory gene expression. The histone-induced expression of CCL4, one of the DEGs with the largest fold change, was demonstrated in THP-1 cells (**Figure 5F**). This result showed that histone toxicity was related to inflammatory and apoptosis pathways and that arginine residues in the H2B-α1 peptide play an important role in histone toxicity.

## Discussion

Extracellular histones are major components of NETs, and their pro-inflammatory effect is known in various inflammatory processes (11, 12, 34, 35); however, the role of extracellular histones in synovial inflammation remains to be elucidated. In this study, we showed that there is an increase in the levels of histones in the synovial fluid of patients with RA compared to those in patients with OA. Previously, it has been demonstrated that the synovial fluid of patients with RA contains a large number of activated neutrophils and macrophages involved in synovial inflammation (26, 27, 36). In a sepsis model, histones released by NETosis trigger innate immune cells, and histones bound to endothelial cells cause endothelial injury (37). Based on the inflammatory nature of NETosis and its resulting extracellular histones, histones from neutrophils in RA synovial fluid would interact with other cells in the joint space, including macrophages and synoviocytes. To validate the pro-inflammatory effect of extracellular histones in arthritis, we demonstrated that intraperitoneal injection of histones aggravated arthritis severity in the STA mouse model.

To confirm the histone-induced cytotoxicity in cells in synovitis, we showed histone-induced cell death in synoviocytes and macrophages through direct treatment of the cells with histones. The results suggested that histones might contribute to synovial inflammation by inducing lytic cell death of synoviocytes and macrophages. Interestingly, time-course dead cell stain images indicated that THP-1 cells are more susceptible to histone-induced cytotoxicity than MH7A cells. Consistent with previous findings, our study demonstrated that histones induce the production of DAMPs, including ATP, HMGB1, and IL-1β, and increase the secretion of TNF-α (38–40). Extracellular ATP released from damaged cells is known to recruit macrophages and promote the secretion of IL-1β by dendritic cells, accelerating the inflammatory process (41). Histone-induced TNF-α production was further augmented by LPS. Therefore, it may have an augmented pro-inflammatory effect in patients with RA.

In addition, we verified that histone-induced cytotoxicity is regulated by electrostatic interaction. It is known that positively charged histones react with the negatively charged cell membrane, thereby causing cytotoxicity (42). Further, pro-inflammatory cytokines such as TNF-α are known to increase surface anionic molecules (43). In our study, LPS increased the binding of positively charged poly-L-lysine on cell surfaces. Furthermore, we found that anionic molecules such as chondroitin sulfate in the synovial fluid could inhibit histone-induced cytotoxicity. In addition, histone-induced cytotoxicity could be mitigated by adding sodium perchlorate, a sulfation inhibitor (44, 45). Considering that sulfation amplifies the anionic nature of proteins, these results suggested that electrostatic conditions of cell surface could regulate histone toxicity by inhibiting or enhancing the binding of histones. To further verify the electrostatic nature of the cytotoxicity of extracellular histones, we modified the cytotoxic α1 peptide of H2B by citrullinating its positively charged arginine residues. The citrullinated H2B-α1 peptide was less cytotoxic to THP-1 cells.

We found that all four classes of recombinant histone proteins induced cell death as the concentration increased, with H2A and H2B being the most cytotoxic. We also showed that H2B-α1 peptide was the most cytotoxic among histone α1 helix peptides from the four classes of histones. This is consistent with previous findings, which showed that H2B derived from frogs have bactericidal properties due to disruption of the bacterial membrane by the cationic nature of its N-terminal region (46, 47).

Using RNA-seq, we have shown that histones changed the expression of specific signaling pathways in THP-1 cells, including IL-1, 4, 10, 13 signaling, chemokines, TLR, and NOD-like receptor (NLR) signaling pathways. The chemokine signaling pathway had several DEGs with the highest degree of fold change, such as CCL4 and CXCL2. It is also known that expression of CCL3, CCL4, CXCL2, and CXCL8 is increased in RA synovial fluid, and that these chemokines are suggested to help neutrophils become resident within the joint in RA (48).

It has been known that NETosis is increased in RA synovium, which induces citrullination of histones by PADs (20, 21). However, there are citrullinated and uncitrullinated parts in extracellular histones derived from the synovial fluids of RA patients (9, 20). Although the hypercitrullinated milieu in RA might reduce the histone-mediated cellular injury (cytotoxicity, DAMP release, chemotaxis), which makes translating our observations into RA pathogenesis less interesting, there are several possibilities that uncitrullinated parts of extracellular histones are relevant to current understanding of RA pathogenesis. First, they might be involved in the breaking of immune tolerance on citrullinated histone during a very early stage of RA. Although citrullination occurs during physiological processes such as apoptosis and NETosis, autoantibody reactivity toward citrullinated antigens is an unusual pathologic process for which the molecular mechanism is unknown. Uncitrullinated parts of extracellular histones may play a role as an adjuvant. Second, they might be involved in local joint inflammation during the established stage of RA. Because there is no association between the ACPA titer and disease activity, the amount of citrullinated antigen is not associated with disease activity. Uncitrullinated parts of extracellular histones may play a role during an acute flare of arthritis. Third, they might be involved in seronegative RA. Seronegative RA patients displayed elevated IgG reactivity to native histone H2B compared to controls, but no citrulline-specific reactivity (49).

The limitations of this study include that the effects of post-translational modification of histones in RA were not fully investigated. Additionally, the influence of anionic proteins such as chondroitin sulfate in synovial fluid that binds to histones and mitigates their cytotoxic effects must be considered in future research. Another limitation is the lack of research into the association between extracellular histones and the formation of anti-citrullinated protein antibodies. Although we established the cytotoxicity and induction of DAMPs by extracellular histones, further research is needed to determine the net effects of extracellular histones on the development of autoimmunity in RA.

In conclusion, our study demonstrated that extracellular histones induce the production of DAMPs, such as ATP, HMGB1, and IL-1β, by causing lytic cell death of synoviocytes and macrophages. Our findings suggest a novel mechanism for the pathogenesis of RA in which extracellular histones play a proinflammatory role in synovitis. Furthermore, our results from charge-altering experiments imply that targeting the electrostatic effect of extracellular histones may be a possible therapeutic option for RA.

## Data availability statement

All data generated or analyzed during this study are included in this article and its supplementary information files. The RNA-seq data presented in the study is deposited in NCBI’s Gene Expression Omnibus (https://www.ncbi.nlm.nih.gov/geo) and Sequence Read Archive (https://www.ncbi.nlm.nih.gov/sra). The data is accessible through GEO accession number GSE205096 (https://www.ncbi.nlm.nih.gov/geo/query/acc.cgi?acc=GSE205096).

## Ethics statement

The studies involving human participants were reviewed and approved by Stanford University School of Medicine Institutional Review Boards. Written informed consent for participation was not required for this study in accordance with the national legislation and the institutional requirements. The animal study was reviewed and approved by the Institutional Animal Care and Use Committee at Pusan National University (Miryang, Korea).

## Author contributions

DS, J-CP, and JS designed the study, and JJ, HK, and JK conducted the experiments and analytical methods. JR, BL, and DS performed the animal experiments and joint histology. JJ, LL, HK, JK, JR, BL, WR, DS, J-CP, and JS discussed and interpreted the data. JJ, LL, HK, JK, JR, and BL made the figures and wrote the manuscript. DS, J-CP, and JS developed the hypothesis, secured funding, coordinated, and directed the project. All authors reviewed and edited the manuscript and had final approval of the submitted manuscript.

## Funding

This study was supported by the Basic Science Research Program (grant number NRF-2021R1F1A1059528 to JS, NRF-2020R1A2B5B01002187 to J-CP, and NRF-2018R1A5A2023879 to DS) through the National Research Foundation of Korea, and funded by the Ministry of Education, Science, and Technology.

## Conflict of interest

The authors declare that the research was conducted in the absence of any commercial or financial relationships that could be construed as a potential conflict of interest.

## Publisher’s note

All claims expressed in this article are solely those of the authors and do not necessarily represent those of their affiliated organizations, or those of the publisher, the editors and the reviewers. Any product that may be evaluated in this article, or claim that may be made by its manufacturer, is not guaranteed or endorsed by the publisher.
