# Supplementary material for: Extracellular histones aggravate autoimmune arthritis by lytic cell death
Source: Front Immunol. 2022 Aug 11;13:961197. doi: 10.3389/fimmu.2022.961197 (PMC9410568; doi:10.3389/fimmu.2022.961197)
Supplement: Supplementary file 1 [file DataSheet_1.docx]

Supplementary Material


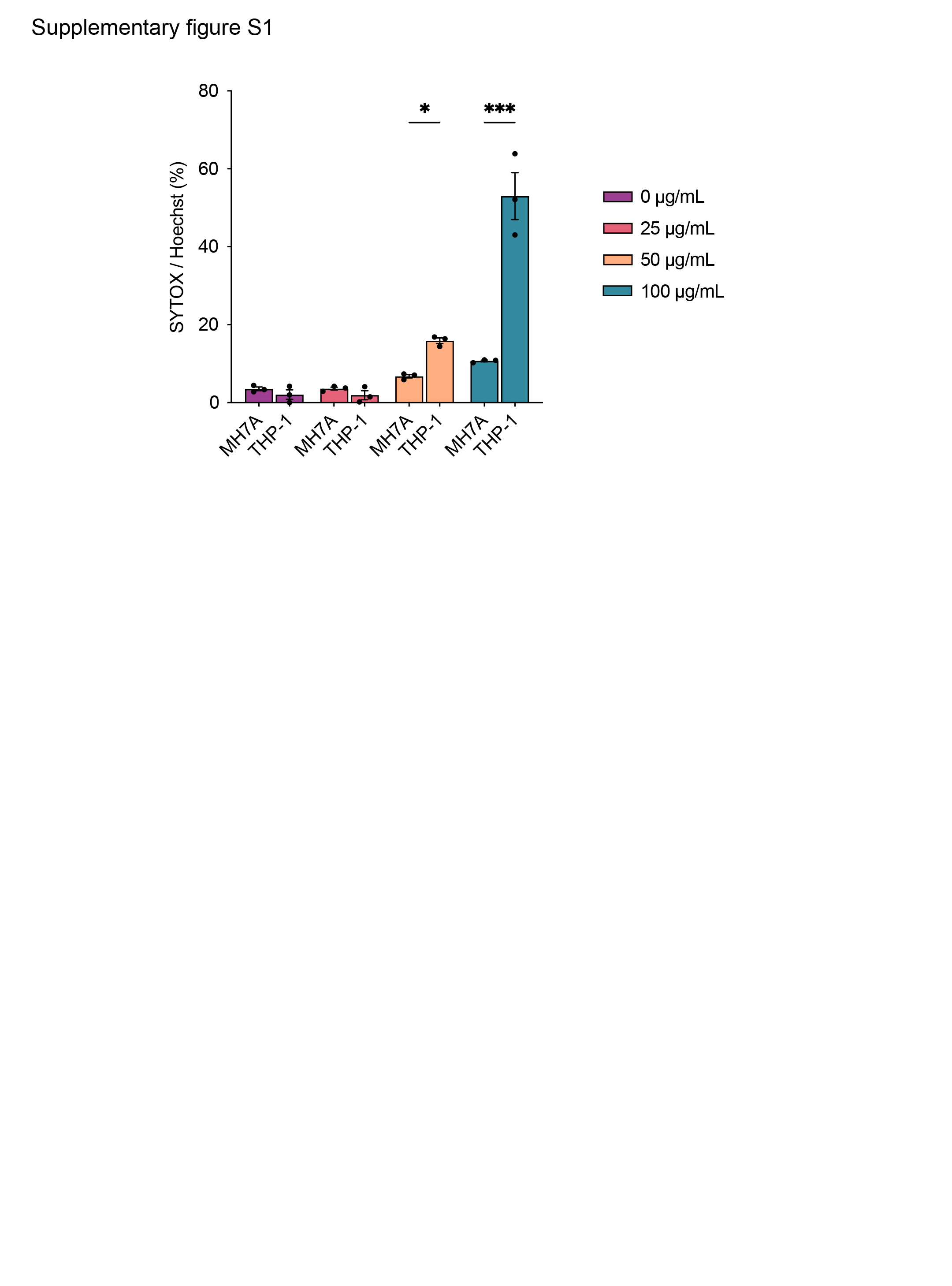


**Supplementary Figure S1.** **SYTOX Green Dead Cell Staining in MH7A and THP-1 cells.**

Analysis of dead cells treated with histones for 1.5 h using SYTOX Green Dead Cell Staining in MH7A and THP-1 cells. Data are expressed as the mean ± standard error of the mean. ANOVA with Tukey–Kramer multiple comparison tests were conducted. **P* < 0.05, ***P* < 0.01, and ****P* < 0.001. The experiments were performed with technical replicates of three wells per condition.


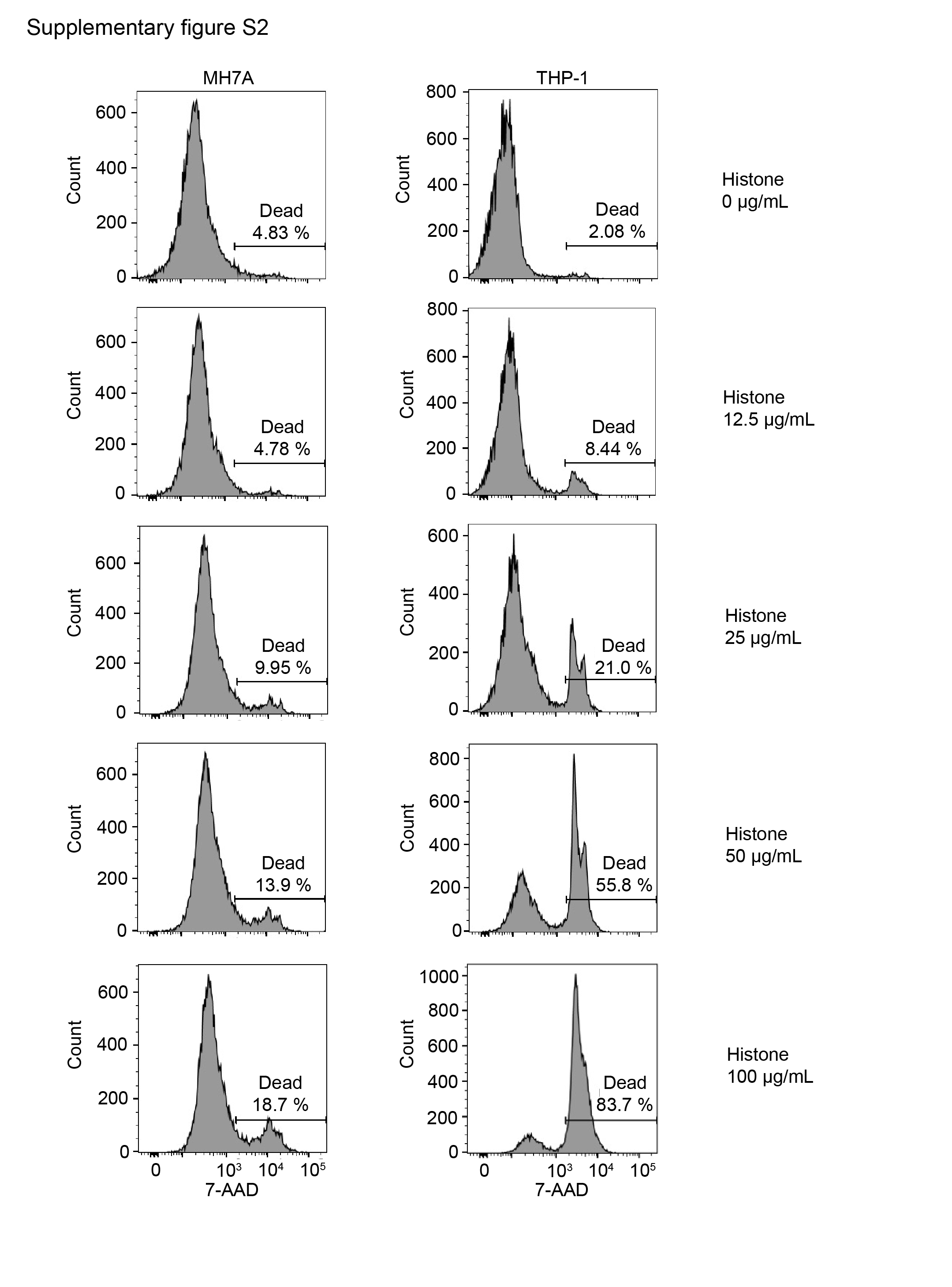
**Supplementary figure S2. Flow cytometry to measure histone-induced cytotoxicity in MH7A and THP-1 cells.**

Analysis of dead cells treated with histones for 1 h using 7-aminoactinomycin D (7-AAD) staining in MH7A (left panels) and THP-1 (right panels) cells.

**Supplementary figure S3. Endotoxin content in the *E. coli*-derived recombinant histones.**

The levels of LPS in 4 μM of histone peptides were determined using a ToxinSensor^TM^ Chromogenic Limulus amebocyte lysate assay kit (GenScript, Piscataway, NJ, USA) according to the manufacturer’s protocols. LPS was detected only in H2B and H3 among all recombinant histones. Data are expressed as the mean ± standard error of the mean. ANOVA with Tukey–Kramer multiple comparison tests were conducted. *P < 0.05, **P < 0.01, and ***P < 0.001. The experiments were performed with technical replicates of three wells per condition.


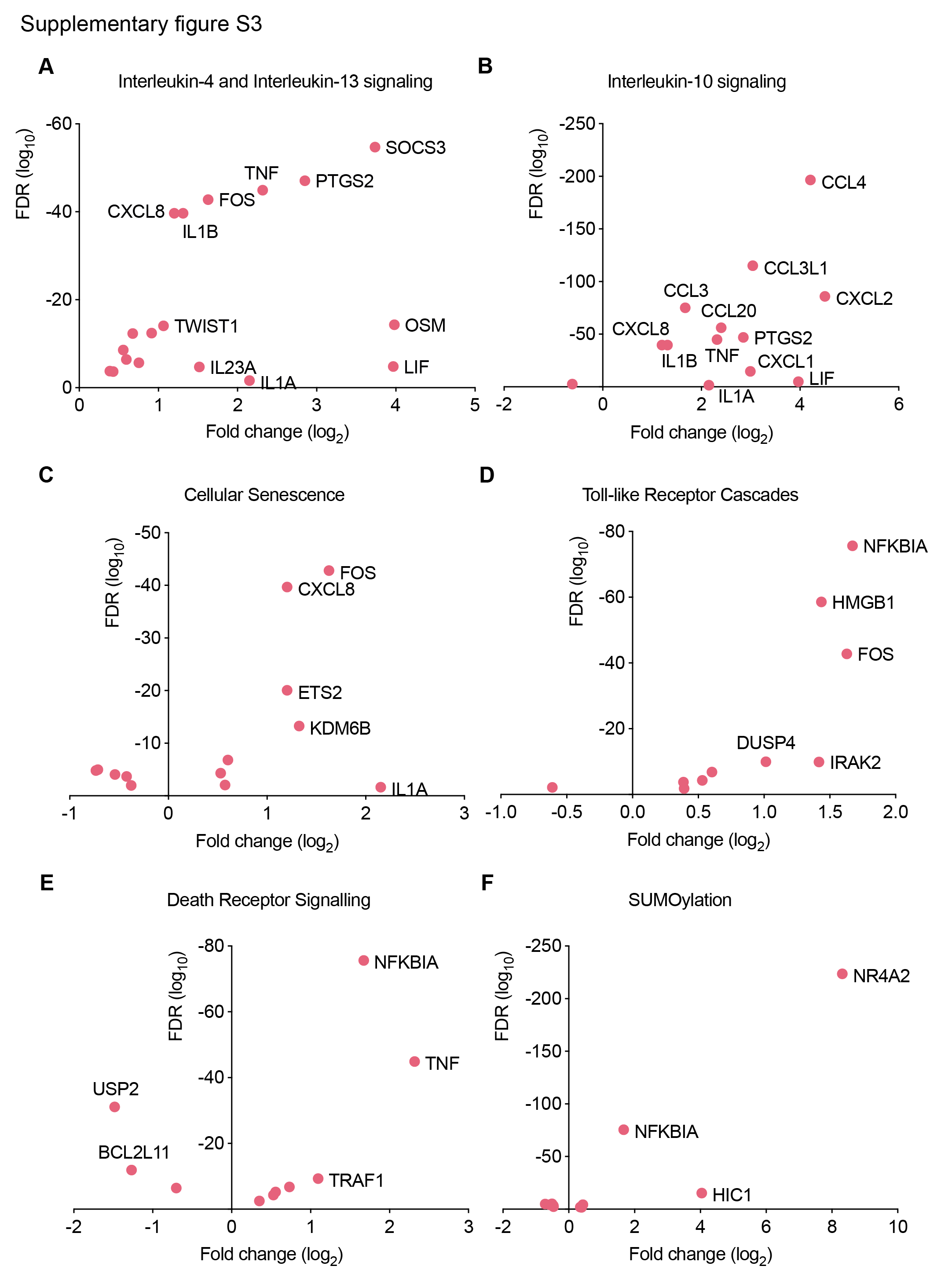


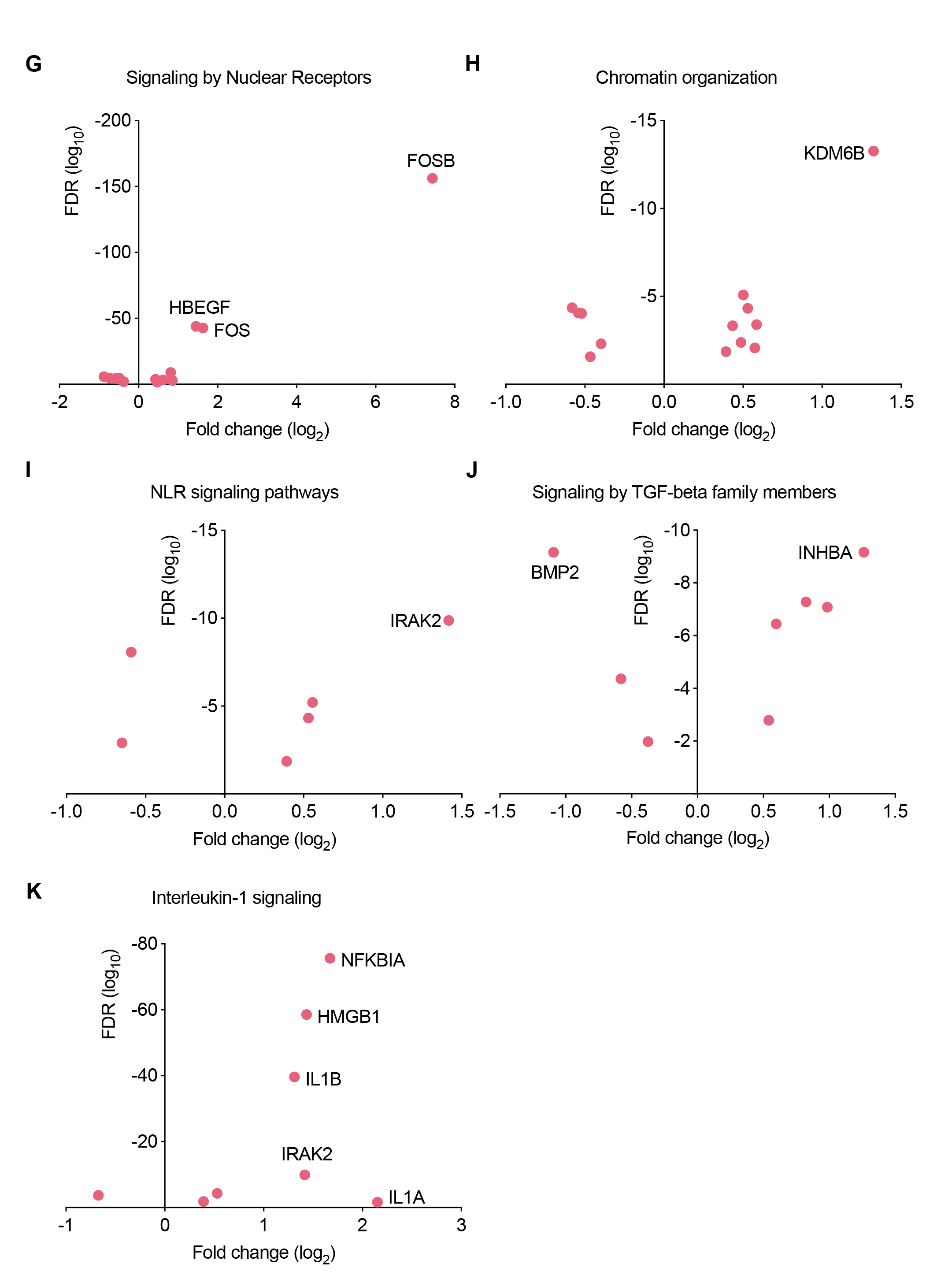


**Supplementary figure S4.** **Significantly enriched Reactome pathways and top differentially expressed genes (DEGs) induced by H2B-α1 peptide in THP-1 cells.**

**(A)**–**(K)** Top DEGs in each significant pathway. Transcriptome analysis was performed using THP-1 cells treated with H2B-α1 peptide or cit-H2B-α1 peptide for 3 h. Significantly enriched Reactome pathways (FDR < 0.05) were selected, and each pathway was shown with the DEGs with a fold change ≥ 2 or ≤ 0.5.

**Supplementary movie 1.** **Histones induce cytotoxicity in synoviocytes and macrophages.** Time-course images of MH7A **(A)** and THP-1 **(B)** cells stained with Hoechst 33343 and SYTOX green obtained by a fluorescence microscope (Eclipse Ti2) after treatment with histones (100 μg/mL). Images were acquired every 3 minutes for 1.5 h.
